# Mitochondria in Health and Disease

**DOI:** 10.3390/cells8070680

**Published:** 2019-07-05

**Authors:** Sarah J. Annesley, Paul R. Fisher

**Affiliations:** Department of Physiology, Anatomy and Microbiology, La Trobe University, Bundoora 3086, Australia

**Keywords:** mitochondria, ROS, mitochondrial dynamics, SIRT, neurological disease

## Abstract

Mitochondria are best known as the sites for production of respiratory ATP and are essential for eukaryotic life. They have their own genome but the great majority of the mitochondrial proteins are encoded by the nuclear genome and are imported into the mitochondria. The mitochondria participate in critical central metabolic pathways and they are fully integrated into the intracellular signalling networks that regulate diverse cellular functions. It is not surprising then that mitochondrial defects or dysregulation have emerged as having key roles in ageing and in the cytopathological mechanisms underlying cancer, neurodegenerative and other diseases. This special issue contains 12 publications—nine review articles and three original research articles. They cover diverse areas of mitochondrial biology and function and how defects in these areas can lead to disease. In addition, the articles in this issue highlight how model organisms have contributed to our understanding of these processes.

## 1. Introduction

Mitochondria are generally believed to be essential for eukaryotic life. They are termed the powerhouses of the cell as they produce most of the energy or ATP required by the cell. The mitochondria have their own genome (mtDNA) which is replicated independently of the host genome. The mtDNA in humans encodes 13 proteins mainly to do with oxidative phosphorylation (OXPHOS). The remaining mitochondrial proteins are encoded by the nuclear genome and are imported into the mitochondria. The two genomes must therefore work together. This special issue comprises 12 publications, nine review articles and three original research articles. They cover diverse areas of mitochondrial function and biology in health and disease, and how models have contributed to our understanding of these processes. Understanding the mechanisms of mitochondrial biology, including morphology and dynamics like fission/fusion and mitophagy, has the potential to lead to the development of effective treatments. 

## 2. Mitochondrial Biology

### 2.1. Repair of mtDNA

Mitochondrial DNA is more susceptible to damage than nuclear DNA, with a 10–20 fold higher rate of mutagenesis than the nuclear genome. This higher mutation rate is due to several factors; the mitochondrial genome contains no introns or histones, it is in close proximity to the sites of reactive oxygen species (ROS) production, it favors a higher amount of dGTP than other dNTPs and its replication follows an asymmetrical division. Apart from the mtDNA mutations associated with severe disease, an accumulation of mtDNA mutations has been associated with aging and age-related diseases such as neurological disorders and with several cancers. One of the main producers of mtDNA damage is lesions due to oxidative damage and these lesions can be repaired by DNA repair pathways. The article by Sharma and Sampath [1] reviews the DNA repair pathways used to maintain mtDNA integrity, focusing on the predominant repair pathway used by the mitochondria called base excision repair (BER) and damage caused by oxidative stress. The article reviews the current literature about these processes and how defects in these pathways can lead to disease progression. Mutations to mitochondrial repair enzymes have been associated with an increased risk of Parkinson’s disease and their levels are altered in several cancers. In addition, defects in the BER pathway have been associated with diabetes and obesity. It is a particularly interesting topic as it opens up another avenue for the treatment of age-related disorders.

### 2.2. ROS Production

The major role of mitochondria are to generate ATP for the cell. This process relies on OXPHOS and a by-product of this process is the generation of ROS. ROS are generated almost entirely by OXPHOS (about 90%). The review by Kausar et al. [2] presents the current literature on how ROS is produced by the mitochondria. Complex I has long been regarded as the main producer of ROS. Likewise, Complex III has been known for some time to be a considerable producer of ROS but more recent literature describes the mechanism of this process and has revealed that ROS are produced by both a forward and reverse electron transfer. Complex II has traditionally not been thought to be a large producer of ROS, but recent evidence has identified that this complex can produce ROS under certain physiological conditions, especially when it is supplied with excess succinate. The evidence for this came from studies in various model systems. The paper then goes on to review the literature linking elevated ROS to disease progression in neurological disorders, namely Huntington’s disease and Parkinson’s disease, where dopamine degradation causes an increase in ROS production. The review highlights the need for further study into the mechanism of ROS production and ponders why strategies aimed at reducing ROS as a treatment option have shown no promise in human clinical trials despite promising results in models.

### 2.3. Mitochondrial Biogenesis

Mitochondrial biogenesis is a complex process requiring coordination of mtDNA and nuclear DNA proteins. There are many homeostatic mechanisms in place to maintain a balance between mitochondrial biogenesis (and the generation of ATP) and excessive production of ROS. In this special issue, this balancing act is discussed. In particular, the article by Bouchez and Devin [3] reviews the role of the cAMP/PKA (cyclic adenosine monophosphate/Protein Kinase A) signaling pathway in ROS production and mitochondrial biogenesis in yeast and in mammals. In yeast, mitochondrial content is tightly regulated by cAMP signalling with an increase in cAMP signalling resulting in increased mitochondrial content. In yeast, biogenesis of mitochondria is regulated by heme activator proteins (HAP) proteins and cAMP signaling was shown to positively regulate HAP proteins and decrease mitochondrial biogenesis. The yeast genome encodes three PKA catalytic subunits named TPK (thiamine pyrophosphokinase) 1–3. Despite functional redundancy in several processes, TPK3 is the only subunit which plays a role in ROS production and consequently mitochondrial biogenesis. TPK3 null mutants displayed an increase in ROS production which degrades Hap4p, reducing HAP complex activity and leading to a reduction in mitochondrial biogenesis. In yeast, ROS production can therefore regulate mitochondrial biogenesis.

In mammals, the cAMP/PKA signaling pathway can regulate OXPHOS activity via its interaction with protein A kinase anchoring proteins (AKAPs) in the outer mitochondrial membrane. PKA activates cAMP response element-binding protein (CREB), which can localize to the mitochondria and initiate transcription of many genes including those involved in mitochondrial biogenesis. The PKA/cAMP pathway is also involved in regulating mitochondrial dynamics, with most evidence favoring its control of fission rather than fusion. ROS levels are linked to mitochondrial biogenesis, yet the direction of this is unclear. The authors present evidence for increased ROS leading to an increase or a decrease in different situations and suggest that the duration of the oxidative stress (acute or chronic) may be the cause of the differing results. Given its importance in health and disease, this is an area worthy of more study.

### 2.4. Mitochondrial Fission

Mitochondrial fission is important for the removal of impaired mitochondria by mitophagy. The review by Zorov et al. [4] presents the literature and experimental evidence that elucidated the process of fission, including the induction of fragmentation by ROS, the proteins involved in induction of division, the scission machinery and the morphological changes to the mitochondria during this process. The mitochondrial shape and volume can be influenced by cytoskeletal and endoplasmic reticulum (ER) interactions, yet these extramitochondrial factors alone cannot explain local mitochondrial swelling events. The authors postulate that an intramitochondrial cytoskeleton may be responsible for responding to these local morphological changes. This idea has been a matter of debate for several decades and the authors present evidence in favor of such an intramitochondrial cytoskeleton and how this structure could be beneficial for physically separating mitochondrial components destined for waste.

### 2.5. Mitochondrial Dynamics

Mitochondrial dynamics is a balance between fission and fusion and the regulation of genes controlling these processes. A shift in the expression levels of one or other type can lead to mitochondrial morphological changes (i.e., fragmentation, spheroid formation etc.). The paper by Hara et al. [5] describes all the different morphological changes that can occur with mitochondria and how the dysregulation of quality control systems such as mitochondrial dynamics and mitophagy is implicated in age-related diseases—specifically two age-related lung diseases, chronic obstructive pulmonary disease (COPD) and idiopathic pulmonary fibrosis (IPF). 

In COPD, the authors discuss how the mechanism is different with acute or chronic exposure to causative agents. Low doses of exposure to cigarette smoke induces elongation of the mitochondria, while more toxic levels induce elongation followed by fragmentation, and longer exposures result in cell death. The authors review the mechanism behind each of the morphological changes including the dysregulation of the quality control systems such as mitochondrial biogenesis and mitophagy. 

In IPF, the lung epithelial cells are exposed to chronic stresses in combination with ageing which itself leads to decreased mitochondrial function and increased stress. This results in increased ER stress and lung fibrosis in IPF. In IPF epithelial cells, there is increased mitochondrial fusion, and this is associated with decreased function. This leads to the production of mitochondria which are less effective at producing ATP and produce higher amounts of ROS. Changes to expression levels of proteins involved in quality control systems such as Parkin (PARK2) and PTEN-induced kinase 1 (PINK1) provide mechanistic insight into how the morphological changes are induced in IPF. This data suggests a decrease in mitophagy, increased fusion and decreased biogenesis. A role for sirtuin 3 (SIRT3) was also predicted. 

### 2.6. SIRT Proteins

Sirtuin proteins are a group of highly conserved proteins present in all domains of life. SIRT3 is a deacetylase enzyme localized to the mitochondria and is important for energy metabolism. Most of the proteins involved in energy metabolism are regulated by acetylation and deacetylation and SIRT3 is the major deacetylase protein of the mitochondria. Mitochondrial proteins in the heart tissue are three times more acetylated than in any other tissue, which enables the cardiac tissue to switch its substrate utilization according to energy demand. Bagul and colleagues [6] present a research article investigating the role of SIRT3 in diabetic cardiomyopathy (DCM), a heart disease often caused by mitochondrial dysfunction. They used a type I diabetic rat model to study the effect of hyperglycemia and lack of insulin on cardiac mitochondrial energetics and transcription. Their study showed that SIRT3 activity was reduced in the diabetic heart and was associated with increased acetylation and reduced activity of mitochondrial transcription factor A (TFAM), a mitochondrial transcription factor. This was followed by reduced activity of electron transport chain complex assembly and ATP levels, as well as decreased cardiac size. The authors treated the rats with resveratrol, which prevented all the cardiac changes and improved cardiac health. Resveratrol directly or indirectly activated SIRT3, leading to reduced acetylation of the mitochondrial transcription factor TFAM and rescued the mitochondrial dysfunction in the diabetic heart. Further elucidating the role of SIRT3 and its substrates and the effects of resveratrol would be beneficial in the development of treatments for heart disease. 

In addition to SIRT3 discussed above, there are several other SIRT proteins in mammalian cells. These different SIRT proteins have some overlapping functions and some independent functions. SIRT1 plays an important role in maintaining a balance between mitochondrial biogenesis and degradation by mitophagy. In this special issue, Song and Hwang [7] investigated the role of SIRT1 in glucose withdrawal which is used as a model to study homeostatic defense mechanisms. Withdrawal of glucose led to an increase of the NAD/NADH (Nicotinamide adenine dinucleotide oxidized/reduced) ratio and the activation of SIRT1 and also to the activation of AMPK (AMP-activated protein kinase). The activation of SIRT1 increased mitochondrial biogenesis and the increased AMPK activity increased the rate of autophagy but not mitophagy. This led to an increased production of ATP due to increased mitochondrial biogenesis. However, the elevated rate of mitochondrial biogenesis exceeded the rate of mitophagy, producing elongated mitochondria with a higher rate of production of ROS and ATP. The role of SIRT1 in glucose withdrawal may provide insight into processes that occur in other conditions of nutrient deprivation such as in calorie restriction or fasting. SIRT1 and other proteins such as SIRT3 have also been shown to be activated in these conditions, but their mode of action and whether the morphological changes to the mitochondria also occur are unknown and warrant further investigation. 

### 2.7. Mitochondrial Proteome and Regulation

The mitochondria participate in a range of functions in addition to its primary role as energy producers. They are important as sites of Ca^2+^ storage and can regulate calcium responses. They can also mediate cell proliferation, differentiation and death, amongst other roles. These numerous essential functions rely on a number of mitochondrial proteins or the mitochondrial proteome which is tightly regulated. The mitochondrial proteome is constantly surveyed for any damaged or misfolded proteins and these proteins are removed largely by the action of AAA (ATPases Associated with diverse cellular Activities) proteases. Mitochondrial AAA proteases couple energy derived from ATP hydrolysis to versatile functions. The article by Opalinska and Janska [8] reviews the literature of AAA proteases in yeast, plants and animals and highlights the numerous roles these proteases play. 

Historically, AAA proteases have been studied for their role in removing damaged or unfolded mitochondrial proteins, yet the current literature shows us that these proteases do much more than this. They are involved in protein quality control, removing aberrant OXPHOS constituents and thereby contributing to optimal formation and maintenance of OXPHOS. They maintain other vital inner membrane (IM) complexes, including the mammalian mitochondrial calcium uniporter (MCU) and mitochondrial cristae organizing system (MICOS). They also mediate the turnover of regulatory proteins modulating key processes, including mitochondrial biogenesis and stress responses. A deficiency in these proteases can result in impairment in mitochondrial translation, disturbances in mitochondrial morphology, calcium deregulation and dysfunction of mitochondrial anterograde transport. All these disturbances can result in cell death. The authors highlight the fact that not all the substrates of these proteases are known and perhaps not all of their functions are known. Given the diverse and important roles identified to date, it will be interesting to see what further research uncovers for these proteases.

## 3. Model Organisms

Model organisms have greatly contributed to our understanding of mitochondrial biology in health and disease. Many of the articles in this issue highlight the contributions of organisms like yeast, plants and rodents and how these organisms could be used to model complex diseases with a mitochondrial involvement like the modelling of Parkinson’s Disease (PD) in *Dictyostelium*. The article by Chernivec et al. [9] modelled PD in *Dictyostelium* by exposure to rotenone and uncovered the importance of mitochondrial dynamics in the disease progression. *Dictyostelium* has long been used as a model for cytoskeletal dynamics, a well characterized process in this organism. Mitochondrial dynamics is composed of fusion, fission and motility. In this study, they measured mitochondrial dynamics in rotenone-treated cells and observed that rotenone disrupted the actin cytoskeleton and microtubule network and this appeared to be independent of the increased ROS. The mitochondrial morphology was unchanged, yet there were significant decreases in fusion events and increased velocity of mitochondrial movement within the cell, but the number of moving mitochondria remained unchanged. These effects were ascribed to changes in the cytoskeleton rather than changes in ROS. Notably, rotenone treatment in *Dictyostelium* does cause increased ROS but no difference to ATP levels as it does in rats. This study highlights how model systems can provide mechanistic insight into complex brain diseases. 

## 4. Disease

As indicated in the previous sections, the mitochondria play essential roles in numerous processes and defects in these processes can lead to disease. In this special issue, the evidence for mitochondrial dysfunction in neurological conditions, metabolic conditions and cancer was reviewed. 

### 4.1. Neurological Disorders

Given the high energy demand of neurons, it is not surprising that mitochondrial defects can have large effects on neurons. The review by Zhou et al. [10] discusses the role of the mitochondria in metabolic dysregulation and disease pathogenesis in several neurological disorders including Alzheimer’s disease (AD), traumatic brain injury (TBI) and epilepsy. Neurons from AD patients display a defect in glucose uptake, and the defect in glucose uptake may precede clinical symptoms, suggesting it may be causal. A mutation in the apolipoprotein E4 (E4) gene is the most common mutation relating to AD. People carrying this mutation display brain glucose hypometabolism, similar to that seen in AD. This is seen even in normal E4 carriers with no dementia and the metabolic deficits are present decades in advance of AD onset in E4-positive and other at-risk individuals, thereby lending support to this being an inherent biological feature of E4 and supporting a causal role in AD.

The authors discuss TBI and provide the evidence for mitochondrial dysfunction in this disorder. There is strong evidence for alteration of Ca^2+^ homeostasis, alteration in amino acids such as glutamate, and increased acute hypoxia. The final neurological disorder they investigate is epilepsy. It is known that mtDNA mutations or mutations to nuclear-encoded mitochondrial proteins can lead to epilepsy. A seizure can result in the release of ROS, an influx of calcium and other neurotransmitter imbalances, which leads to further disruption of mitochondrial metabolism. The evidence to date has not uncovered whether mitochondrial dysfunction is a cause or consequence or perhaps both, yet mitochondrial dysfunction is clearly involved in the pathogenesis of epilepsy.

### 4.2. Cancer

Cancer cells are generally thought to be more reliant on glycolysis for energy production rather than OXPHOS. Salazar et al. [11] used STRING (Search Tool for the Retrieval of Interacting Genes/Proteins) and other databases to analyze the relationship of common PD and carcinogenic proteins PARKIN, PINK1 and DJ-1 (Protein deglycase). The three proteins have roles in mitochondrial morphology and regulate mitochondrial quality control. The STRING database and gene ontology (GO) enrichment analysis were used to consolidate knowledge of well-known protein interactions for PINK1, PARKIN, and DJ-1 and envisage new ones. Some predicted transcription factors regulating PINK1, PARK2 (PARKIN) and PARK7 (DJ-1) gene expression were related to cell cycle control. This suggests that the interplay among the PINK1/PARKIN/DJ-1 network during mitochondrial quality control in cancer biology may occur at the transcriptional level.

### 4.3. Fatty Acid Oxidation (FAO) Disorder ECHS1 (Short-Chain Enoyl-CoA Hydratase):

When glucose supply is decreased, this can lead the cells to increase their oxidation of fatty acids as alternative substrates for mitochondrial energy. Deficiencies in fatty acid oxidation (FAO) can lead to very serious metabolic disease in early childhood, mainly affecting tissues which are more dependent on FAO for metabolism such as the heart, liver and skeletal muscle. Unlike most FAO disorders, a deficiency in the FAO enzyme short-chain enoyl-CoA hydratase (ECHS1), which is a key FAO enzyme involved in the metabolism of fatty acyl-CoA esters, leads to a form of Leigh disease. Leigh disease is traditionally associated with defects in OXPHOS. The article by Sharpe and McKenzie [12] reviews the 40 patients which have been identified with this disease since the first case described in 2014. It identifies similarities between the cases and the severity of the disorder. A defect in FAO and valine metabolism is evident in most cases and secondary OXPHOS defects have been identified in patients with a more severe phenotype. The paper highlights the complex interaction between FAO and OXPHOS.

## 5. Final Remarks

The reviews and research articles in this special issue illustrate the intimate roles that mitochondria play in almost all aspects of cellular function. This close interaction between mitochondria and the rest of the cell is the reason why mitochondria and their dysregulation participate in the cytopathological processes underlying such diverse diseases as cancer, neurological diseases and metabolic diseases. The articles in this special issue provide a fascinating snapshot of many of these roles in mitochondrial biology and disease.
